# Lactate modulates microglia polarization via IGFBP6 expression and remodels tumor microenvironment in glioblastoma

**DOI:** 10.1007/s00262-022-03215-3

**Published:** 2022-06-03

**Authors:** Lucia Longhitano, Nunzio Vicario, Stefano Forte, Cesarina Giallongo, Giuseppe Broggi, Rosario Caltabiano, Giuseppe Maria Vincenzo Barbagallo, Roberto Altieri, Giuseppina Raciti, Michelino Di Rosa, Massimo Caruso, Rosalba Parenti, Arcangelo Liso, Federica Busi, Marco Lolicato, Maria Caterina Mione, Giovanni Li Volti, Daniele Tibullo

**Affiliations:** 1 Department of Biomedical and Biotechnological Sciences, University of Catania, Catania, Italy; 2 IOM Ricerca, 95029 Viagrande, CT Italy; 3 Department of Medical and Surgical Sciences and Advanced Technologies, F. Ingrassia, Anatomic Pathology, University of Catania, Catania, Italy; 4 Department of Drug Sciences, University of Catania, Catania, Italy; 5 Department of Medical and Surgical Sciences, University of Foggia, 71100 Foggia, Italy; 6 Department of Cellular, Computational and Integrative Biology Cibio, University of Trento, 38123 Trento, Italy; 7 Department of Molecular Medicine, University of Pavia, Pavia, Italy

**Keywords:** Glioblastoma, IGFBP6, Microglia, Lactate, Microenvironment

## Abstract

Lactic acidosis has been reported in solid tumor microenvironment (TME) including glioblastoma (GBM). In TME, several signaling molecules, growth factors and metabolites have been identified to induce resistance to chemotherapy and to sustain immune escape. In the early phases of the disease, microglia infiltrates TME, contributing to tumorigenesis rather than counteracting its growth. Insulin-like Growth Factor Binding Protein 6 (IGFBP6) is expressed during tumor development, and it is involved in migration, immune-escape and inflammation, thus providing an attractive target for GBM therapy. Here, we aimed at investigating the crosstalk between lactate metabolism and IGFBP6 in TME and GBM progression. Our results show that microglia exposed to lactate or IGFBP6 significantly increased the Monocarboxylate transporter 1 (MCT1) expression together with genes involved in mitochondrial metabolism. We, also, observed an increase in the M2 markers and a reduction of inducible nitric oxide synthase (iNOS) levels, suggesting a role of lactate/IGFBP6 metabolism in immune-escape activation. GBM cells exposed to lactate also showed increased levels of IGFBP6 and vice-versa. Such a phenomenon was coupled with a IGFBP6-mediated sonic hedgehog (SHH) ignaling increase. We, finally, tested our hypothesis in a GBM zebrafish animal model, where we observed an increase in microglia cells and *igfbp6* gene expression after lactate exposure. Our results were confirmed by the analysis of human transcriptomes datasets and immunohistochemical assay from human GBM biopsies, suggesting the existence of a lactate/IGFBP6 crosstalk in microglial cells, so that IGFBP6 expression is regulated by lactate production in GBM cells and in turn modulates microglia polarization.

**Supplementary Information:**

The online version contains supplementary material available at 10.1007/s00262-022-03215-3.

## Introduction

Glioblastoma Multiforme (GBM) is the most common primary brain tumor, in which infiltration pattern, radioresistance and recurrences are among the most critical limiting factors in developing effective therapies [1]. Treatment resistance, tumor recurrence and poor prognosis are the combined results of both cancer cell proliferation and the interaction with tumor microenvironment (TME) [2] which includes different cell types such as glioma-associated microglia/macrophages (GAMs).

Microglia are sentinels cells of the central nervous system (CNS), interacting with astrocytes and other cell populations and playing key roles under both physiological and pathological conditions [3]. GAMs are functionally similar to that of tumor-associated macrophages in the peripheral system and closely interact with GBM cells via intracellular communications [4]. Although GAMs possess a few innate immune functions, their ability to be stimulated via toll-like receptors (TLRs), secreting cytokines, and upregulating co-stimulatory molecules is not sufficient to initiate antitumor immune responses [5]. However, in malignant gliomas, M2-polarization of microglia leads to an immunosuppressive and tumor-supportive phenotype triggered by a series of tumor cytokines, such as transforming growth factor-β, interleukin-10, and prostaglandin E2 and other growth factors [6].

TME is critical to establish malignancy and it is characterized by high glycolytic metabolism with increased lactate production [7]. Lactate is largely produced within the TME and is used as an energy-rich substrate, signaling molecule and as an important immune suppressor by tumors. The glycolytic cancer cells and cancer-associated fibroblasts (CAFs) are the main producers of lactate, simply because they are the most abundant populations within the neoplasm [7]. TME enforces to metabolic adaptability, physical pressure, oxidative stress, nutrient deprivation and competition, immune surveillance as well as adaptability to hypoxic and acidic environment, all having an enormous impact on tumor malignancy [8]. Therefore, a high rate of aerobic glycolysis (glucose metabolism) efflux of resultant lactic acid [9], and concomitant acidification of the TME are hallmarks of several cancers, including GBM [10]. Tumor-derived lactate is taken up by GAMs through their monocarboxylate transporters (MCT1, MCT2 and MCT4), leading to the transcription of the vascular endothelial growth factor (VEGF) and the l-arginine- metabolizing enzyme arginase-1 (ARG1) genes inhibiting T-cell activation and proliferation [11]. Furthermore, MCT-mediated H^+^ efflux increases extracellular acidification supporting the formation of a unfavorable environment in which cancer cells that have adapted to these conditions can outcompete normal cells and thus further enhancing tumor progression [12]. Over-expression of lactate transporters is a common feature of cancers with high metabolic rate [13]. To this regard, high expression of MCT1, MCT4 and its chaperone CD147 is associated with decreased progression-free survival in clear cell renal cell carcinoma, head and neck cancers and neuroblastoma [14].

The insulin-like growth factor (IGF) system is ubiquitously present and includes the type I and type II IGF receptors (IGF-I and IGF-II) and specific insulin-like growth factor-binding proteins (IGFBPs), which are a family of six proteins functioning as transport proteins for IGF-I and IGF-II in the circulation and regulating their access to the potentially oncogenic IGF-I receptor (IGF1R) [15]. Interestingly, IGFBPs may inhibit and/or enhance IGF-I and IGF-II biological effects. In particular, the insulin-like growth factor binding protein 6 (IGFBP6) is expressed in a variety of tissues and its expression is developmentally regulated [16] and is characterized by its high IGF-II binding specificity. Several studies showed that IGFBP6 may exert biological effects independently from IGF-II [17], such as regulation of proliferation, apoptosis, angiogenesis and cell migration, suggesting a major role in immunity and in inflammation. Among various growth factors, IGFBP6 was reported to play an important role in survival and migration of tumor cells [18], but its effects on tumor and immune system interaction are still poorly understood and the relationships between IGFBP6 and cancer prognosis remain contradictory in many studies [19].

To this regard, the aim of the present study was to evaluate the crosstalk between lactate and IGFBP6 in microglial cells and how such interaction modulates TME and GBM progression.

## 2. Material and methods

### Cell culture, pharmacological treatments and MTT turnover

Human glioblastoma cell lines (U-87 MG, A-172 and U-251 MG) were purchased from ATCC Company (Milan, Italy). Cells were suspended in DMEM (Gibco, cat. no. 11965092) culture medium containing 10% fetal bovine serum (FBS, Gibco, cat. no. 10082147), 100 U/mL penicillin and 100 U/mL streptomycin (Gibco, cat. no. 15070063). At 80% confluency, cells were passaged using trypsin–EDTA solution (0.05% trypsin and 0.02% EDTA, Gibco, cat. no. 25300054).

Human microglia cell line (HMC3) was purchased from ATCC Company (Milan, Italy). HMC3 cells were cultured according to the recommendations by ATCC, where EMEM (ATCC® 30–2003TM) was used as the base medium and completed by adding 56 mL FBS (ATCC® 30–2020TM) to a 500 mL of base EMEM. Lactate and IGFBP6 (Sigma-Aldrich, Milan, Italy) were added to cell culture of all experiments at final concentrations of 20 mM and 400 ng/mL, respectively, for 24, 48 and 72 h, as previously reported [20]. Cyclopamine was used at a final concentration of 1 μM, as previously reported [21]. The MTT assay was performed as previously described [22], on separate plates. Briefly, a solution of MTT at a final concentration of 5 mg/mL was added to each well and incubated for 2 h at 37 °C/5% CO_2_. Media were then gently removed, MTT solvent (DMSO, Sigma) was added, and cells were stirred on an orbital shaker for 5 min at room temperature. The absorbance was measured using a Varioskan Flash spectrophotometer (Thermo Scientific, Milan, Italy) at 550 nm. Results were expressed as the percentage of MTT reduction versus control cells.

### Real-Time PCR for gene expression analysis

RNA was extracted by Trizol® reagent (category no. 15596026, Invitrogen, Carlsbad, CA, USA). The first-strand cDNA was then synthesized with High-Capacity cDNA Reverse Transcription kit (category no. 4368814, Applied Biosystems, Foster City, CA, USA). High cDNA quality was checked, taking into consideration the housekeeping gene Ct values. Quantitative real-time PCR was performed in Step-One Fast Real-Time PCR system, Applied Biosystems, using the SYBR Green PCR MasterMix (category no. 4309155, Life Technologies, Monza, Italy). The specific PCR products were detected by the fluorescence of SYBR Green, the double-stranded DNA binding dye. Primers were designed using BLAST® (Basic Local Alignment Search Tool, NBCI, NIH), considering the shortest amplicon proposed: primers′ sequences are shown in Table 1, and *β*-actin was used as the housekeeping gene. Primers were purchased by Metabion International AG (Planneg, Germany). The relative mRNA expression level was calculated by the threshold cycle (Ct) value of each PCR product and normalized with *β*-actin by using a comparative 2^−*ΔΔ*Ct^ method.

**Table 1:** List of qRT-PCR primers

Gene of interest	Forward primer (5′ ⟶ 3′)	Reverse primer (5′ ⟶ 3′)
SLC16A1	TGTTGTTGCAAATGGAGTGT	AAGTCGATAATTGATGCCCATGCCAA
SLC16A3	TATCCAGATCTACCTCACCAC	GGCCTGGCAAAGATGTCGATGA
GFBP6	CCTGCTGTTGCAGAGGAGAAT	CTCTGCGGTTCACATCCTGT
PGC1*α*	ATGAAGGGTACTTTTCTGCCCC	GGTCTTCACCAACCAGAGCA
TFAM	CCGAGGTGGTTTTCATCTGT	AGTCTTCAGCTTTTCCTGCG
ATP5F1A	CCGCCTTCCGCGGTATAATC	ATGTACGCGGGCAATACCAT
COX IV	GCGGTGCCATGTTCTTCATC	GGGCCGTACACATAGTGCTT
COX II	GAACTATCCTGCCCGCCATC	AGGGATCGTTGACCTCGTCT
CYTB	TCTTGCACGAAACGGGATCA	TGATTGGCTTAGTGGGCGAA
ND4	ACAAGCTCCATCTGCCTACGACAA	TTATGAGAATGACTGCGCCGGTGA
ARG1	TCACCTGAGCTTTGATGTCG	CTGAAAGGAGCCCTGTCTTG
CD206	CAAGGAAGGTTGGCATTTGT	CCTTTCAGTCCTTTGCAAGC
CD163	TCCACACGTCCAGAACAGTC	CCTTGGAAACAGAGACAGGC
TNF	AGAAGTTCCCAAATGGCCTC	CCACTTGGTGGTTTGCTACG
LDHA	GGATCTCCAACATGGCAGCCTT	AGACGGCTTTCTCCCTCTTGCT
ENO1	AAAGCTGGTGCCGTTGAGAAG	AGCATGAGAACCGCCATTGAT
HK2	ATGAGGGGCGGATGTGTATCA	GGTTCAGTGAGCCCATGTCAA
IL1β	CTGGTGTGTGACGTTCCCATTA	CCGACAGCACGAGGCTTT
TLR4	AAGCCGAAAGGTGATTGTTG	CTGAGCAGGGTCTTCTCCAC
SHH	GCGAGATGTCTGCTGCTAGT	TTACACCTCTGAGTCATCAGC
*β*-actin	CCTTTGCCGATCCGCCG	AACATGATCTGGGTCATCTTCTCGC

### Western blot analysis

Briefly, for western blot analysis, 30 μg of protein was loaded onto a 12% polyacrylamide gel MiniPROTEAN® TGXTM (BIO-RAD, Milan, Italy) followed by electrotransfer to nitrocellulose membrane TransBlot® TurboTM (BIO-RAD, Milan, Italy) using TransBlot® SE Semi-Dry Transfer Cell (BIO- RAD, Milan, Italy). Subsequently, membrane was blocked in Odyssey Blocking Buffer (Licor, Milan, Italy) for 1 h at room temperature. After blocking, membrane was three times washed in phosphate-buffered saline (PBS) for 5 min and incubated with primary antibodies against MCT1 (1:1000), MCT4 (1:1000), IGFBP6 (1:500) and β-actin (1:1000) (anti-mouse, Cat. No. 4967S, Cell Signalling Technology, Milan, Italy), overnight at 4 °C. Next day, membranes were three times washed in PBS for 5 min and incubated with infrared anti-mouse IRDye800CW (1:5000) and anti-rabbit IRDye700CW secondary antibodies (1:5000) in PBS/0.5% Tween-20 for 1 h at room temperature. All antibodies were diluted in Odyssey Blocking Buffer. The blots were visualized using Odyssey Infrared Imaging Scanner (Licor, Milan, Italy), and protein levels were quantified by densitometric analysis. Data were normalized to β-actin expression.

### Immunocytochemical analysis

HMC3 cells were grown directly on coverslips before immunofluorescence and treated with lactate at the final concentration of 20 mM and with IGFBP6 at the final concentration of 400 ng/mL for 72 h. After washing with PBS, cells were fixed in 4% paraformaldehyde (category no. 1004968350 Sigma-Aldrich, Milan, Italy) for 20 min at room temperature. Subsequently, cells were incubated with primary antibody against ARG1 and iNOS at dilution 1:200, overnight at 4 °C. The next day, cells were washed three times in PBS for 5 min and incubated with secondary antibodies: TRITC (anti-goat, Santa Cruz Biotechnology, Santa Cruz, CA, USA) at dilution 1:200 for 1 h at room temperature. Cells were washed three times in PBS for 5 min and incubated with phalloidin at dilution 1:500 for 30 min. The slides were mounted with medium containing DAPI (4′,6- diamidino-2phenylindole, category no. sc-3598, Santa Cruz Biotechnology, Santa Cruz, CA, USA) to visualize nuclei. The fluorescent images were obtained using a Zeiss Axio Imager Z1 microscope with Apotome 2 system (Zeiss, Milan, Italy). As a control, the specificity of immunostaining was verified by omitting incubation with the primary or secondary antibody. Immunoreactivity was evaluated considering the signal-to-noise ratio of immunofluorescence.

### Lactate concentration measurement

The spectrophotometric determination of lactate was carried out using an Agilent 89090A spectrophotometer (Agilent Technologies, Santa Clara Ca, USA) and following the method described by Artiss et al. [23]. Briefly, the reaction mixture contained 100 mM Tris–HCl, 1.5 mM N-ethyl-N-2-hydroxy-3-sulfopropyl-3-methylalanine, 1.7 mM 4-aminoantipyrine, and 5 IU horseradish peroxidase. Fifty microliters of serum were added to the mixture, let to stand for 5 min and read at 545 nm wavelength. The reaction was started with the addition of 5 IU of lactate oxidase to the cuvette (finale volume = 1 ml) and it was considered ended when no change in absorbance was recorded for at least 3 min. To calculate lactate in samples, the difference in absorbance at 545 nm wavelength (*Δ*abs) of each sample was interpolated with a calibration curve obtained by plotting *Δ*abs measured in standard solutions of lactate with increasing known concentrations.

### IGFBP6 ELISA test

Cell culture supernatant collected on 24 h from cell-laden hydrogel were frozen at − 80 °C until use. We determined in culture media the quantitative concentrations of IGFBP6 with IGFBP6 Human ELISA Kit (catalog #EHIGFBP6, Invitrogen) according to the manufacturer’s instructions.

### Cell migration

Cell proliferation was studied by employing the “wound healing” assay. Cells were seeded separately in 6-well dishes and cultured until confluence. Cells were scraped with a 200-μl micropipette tip and monitored at 0 h, 24 h and 48 h. The uncovered wound area was measured and quantified at different intervals with ImageJ 1.37 V (NIH). The experiments were done in quadruplicates.

### Clonogenic assay

Colony assays performed by seeding cells in 6-well plates at low density (5000 cells/well) and allowing growth for 10 days. Colonies were fixed, stained with crystal violet and colonies were quantified with Operetta high content screening (HCS) System (Perkin Elmer). The experiments were performed in quadruplicates.

### Zebrafish model

Adult zebrafish (Danio rerio) were housed in the Model Organism Facility–Center for Integrative Biology (CIBIO) University of Trento and maintained under standard conditions [24]. All zebrafish studies were performed according to European and Italian law, D.Lgs. 26/2014, authorization 148/2018-PR to M. C. Mione. Fishes with somatic and germline expression of oncogenic HRAS were generated as described [25, 26]. Two days postfertilization (dpf) were treated daily with 20 mM lactate dissolved in fish water, until 5dpf when analyses of gene expression and microglia were performed; controls were left untreated.

The following zebrafish transgenic lines were used in the course of this study:

- *Et(zic4:Gal4TA4, UAS:mCherry)*_*hzm5*_ called zic:Gal4 [25]
- *Tg(UAS:eGFP-HRAS_G12V)*_*io006*_ called UAS:RAS [26]

The characterization of the GBM model is described in detail in Mayrhofer et al. [25].

### Gene expression analysis

For gene expression analysis, total RNA was extracted from 20 5dpf zic:RAS larvae treated or not with 20 mM lactate, using the NucleoSpin RNA isolation kit (MN) following the manufacturer’s instructions and treated with DNaseI (Thermo Fisher, 10 units/RNA sample). The RNA concentration was quantified using nanodrop2000 (Thermo Fisher) and SensiFAST cDNA Synthesis Kit (Bioline) was used for First-strand cDNA synthesis according to the manufacturer’s protocol. qRT-PCR analysis was performed using ExcelTaq qPCR Master Mix (SMOBIO) using a standard amplification protocol. The primers used for zebrafish *igfbp6a* were: forward 5′‐TGCTTTAGGGCTCCGTTGTA‐3′ and reverse 5′‐ACATGGACCCTTCTCGTTGT‐3′; for zebrafish *igfbp6b* were: forward 5′-TTGTATCCTCTGGCACTGCA‐3′ and reverse 5′-CAGACACCAGGATCCACAGT-3′; for zebrafish *rps11* (housekeeping): forward: 5′‐ACAGAAATGCCCCTTCACTG-3′ and reverse: 5′‐GCCTCTTCTCAAAACGGTTG‐3′. Real-time PCR was performed with a CFX96 Real-Time PCR Detection System (Bio-Rad) machine. Q-PCR analysis was performed with Microsoft Excel and GraphPad Prism. In all cases, each PCR was performed with triplicate samples and repeated with at least three biological replicates.

### Immunofluorescence for microglia markers in zebrafish

For whole-mount immunofluorescence of 5 day postfertilization (dpf) zebrafish, zic:RAS larvae were culled by anesthetic overdose, fixed in 4% PFA for 2 h to 12 h at 4 C, and their brains carefully were removed under a stereomicroscope and processed with the primary antibody diluted in PBS containing 5% normal goat serum and 0.1% triton x-100 at 4 °C overnight. The antibody used and its dilution are: L-plastin (a gift of Prof. Paul Martin, Bristol University), diluted 1:1000 in 5% NGS, 0.5% Triton X100 in PBS overnight. A secondary antibody conjugated with Alexa 633 was used for 12 h at 4 °C. Images were acquired using an inverted Leica TSP8 confocal microscope, after equilibrating the brains in 100% glycerol. Counts were performed manually on 5 different brains for each condition using Image J software.

### Glioblastoma biopsies

Formalin-fixed and paraffin-embedded tissue specimens from 10 patients affected by GBM were obtained from the surgical pathology files at the Anatomic Pathology, Department G.F. Ingrassia, University of Catania, Catania, Italy. Multiple sections (at least 5) were obtained from formalin-fixed and paraffin-embedded tissue specimens. Due to the retrospective nature of the study, no written informed consent from patients was obtained. The study included 6 male and 4 female patients (mean age: 61 years; age range: (41–81). According to the World Health Organization criteria, the histologic diagnosis of GBM was rendered in presence of the following morphological criteria: (i) high-grade glioma with astrocytic morphology; (ii) diffuse growth pattern; (iii) foci of necrosis and/or microvascular proliferation.

### Immunohistochemical analysis

Sections were processed as previously described [27]. Then, the sections were incubated overnight at 4 °C with rabbit polyclonal anti-IGFBP6 antibody (Sigma, Milan, Italy), ready to use in PBS (Sigma, Milan, Italy) and MIB-1, a monoclonal antibody directed against the Ki-67 antigen (M7240; Dako Corporation, Glostrup, Denmark), diluted 1:75 in PBS (Sigma, Milan, Italy). The secondary antibody, biotinylated anti-rabbit antibody was applied for 30 min at room temperature, followed by the avidin–biotin–peroxidase complex (Vector Laboratories, Burlingame, CA, USA) for a further 30 min at room temperature. The immunoreaction was visualized by incubating the sections for 4 min in a 0.1% 3,3′‐diaminobenzidine (DAB) and 0.02% hydrogen peroxide solution (DAB substrate kit, Vector Laboratories, CA, USA). The sections were lightly counterstained with Mayer’s hematoxylin (Histolab Products AB, Göteborg, Sweden) mounted in GVA mountant (Zymed Laboratories, San Francisco, CA, USA) and observed with a Zeiss Axioplan light microscope (Carl Zeiss, Oberkochen, Germany). IGFBP6 staining (both nuclear and cytoplasmic staining) was semi-quantitatively evaluated according to a 0 to 3 scale of Intensity of Staining (IS) and to the percentage of positively stained cells (Extent Score, ES; on a five-tiered system: < 5; 5–30; 31–50; 51–75; > 75%. The immunoreactivity score (IRS) has been obtained by multiplying IS and ES: low (L-IRS) and high (H-IRS) expression of IGFBP6 were respectively defined as IRS < 6 and IRS ≥ 6.

MIB-1 immunohistochemical expression was assessed as low if positive in less than 50% of neoplastic cells, as high if positive in more than 50% of neoplastic cells.

## Transcriptome analysis

### Dataset selection

The NCBI Gene Expression Omnibus (GEO) database (http://www.ncbi.nlm.nih.gov/geo/) [28] was used to select transcriptomes datasets of interest. Mesh terms, “human”, “glioblastoma”, and “tumor grade”, were used to identify the datasets. We sorted the datasets by the number of samples (High to Low), age and sex of the participants and by the clinical data made available by the authors. We selected the GSE108474 dataset [29] over the others available for the number of subjects recruited (541), for the availability of clinical data (tumor staging) and for the variety of tumors analyzed (glioblastoma, oligodendroglioma, astrocytoma and normal subjects).

### Data processing, experimental design and statistics

To process and identify Significantly Different Expressed Genes (SDEG) within the datasets, we used the MultiExperiment Viewer (MeV) software (The Institute for Genomic Research (TIGR), J. Craig Venter Institute, La Jolla, USA). In cases where multiple genes probes have insisted on the same GeneID NCBI, we used those with the highest variance.

For GSE108474, we performed a statistical analysis with GEO2R, applying a Benjamini & Hochberg (False discovery rate, Table 2) [30].

**Table 2:** Samples selected from GSE108474

Disease type	Number	Grade
NT	28	Negative
Astrocytoma	148	NA = 25; G2 = 65; G3 = 58
Oligodendroglioma	67	NA = 14; G2 = 30; G3 = 23
Glioblastoma	221	Na = 91; G4 = 130

*G* tumor grade, *NA* not assigned, *NT* non tumor

Significant differences between groups were assessed using the Ordinary one-way ANOVA test, and Tukey’s multiple comparisons test correction was performed to compare data between all groups. Correlations were determined using Pearson correlation. All tests were two-sided and significance was determined at adjusted p value 0.05. The dataset selected was transformed for the analysis in Z-score intensity signal. Z-score is constructed by taking the ratio of weighted mean difference and combined standard deviation according to Box and Tiao [31]. The application of a classical method of data normalization, z-score transformation, provides a way of standardizing data across a wide range of experiments and allows the comparison of microarray data independent of the original hybridization intensities. The z-score is considered a reliable procedure for this type of analysis and can be considered a state-of-the-art method, as demonstrated by the numerous bibliography [32].

The efficiency of each biomarker across the different tumor grade was assessed by the receiver operating characteristic (ROC) curve analyses [33]. The ROC curves analyzed brain biopsies of healthy subjects (NT) vs glioblastoma patients, astrocytoma vs glioblastoma, and oligodendroglioma vs glioblastoma. The area under the ROC curve (AUC) and its 95% confidence interval (95% CI) indicate diagnostic efficiency.

### Statistical analysis

For statistical analyses, a two-tailed unpaired Student’s t test was used for comparison of *n* = 2 groups. For comparison of *n* ≥ 3 groups, one-way analysis of variance (ANOVA) followed by Bonferroni post-hoc test for multiple comparisons was used. Data are presented as the mean ± *SD*. A value of *P* < 0.05 was considered statistically significant and symbols used to indicate statistical differences are described in figure legends.

## Results

### Lactate induces the expression of MCT1 promoting an oxidative metabolism in microglia cells

**Fig. 1:**
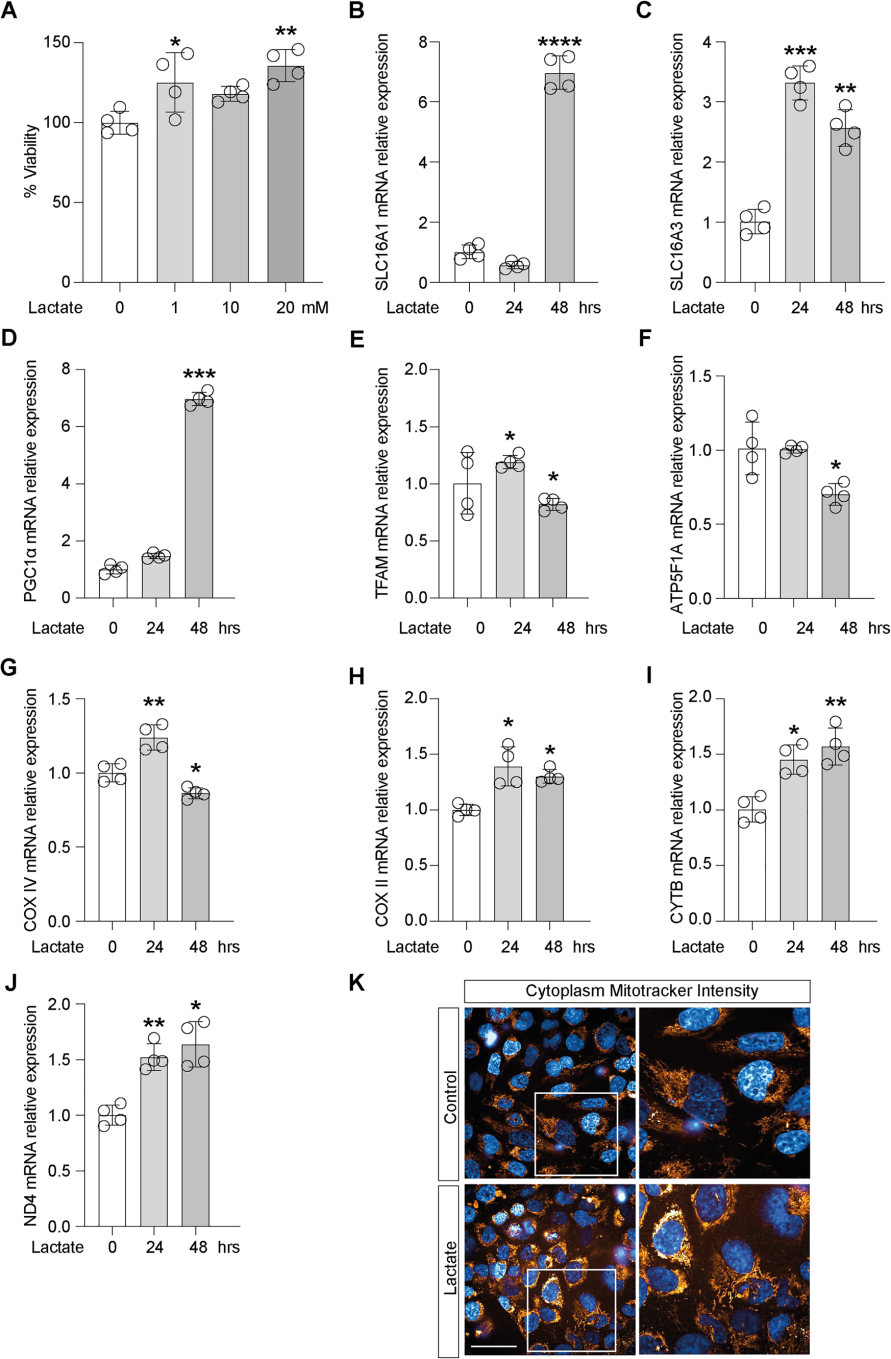
Lactate induces the expression of MCT1, promoting an oxidative metabolism in microglia cells**.** Effect of Lactate on **A** cell viability, **B** *SLC16A1 (MCT1)* and **C** *SLC16A3* (*MCT4)* mRNA expression levels, in microglia cells. Evaluation of relative mRNA expression levels of **D** *PGC1a*, **E** *TFAM*, **F** *ATP5F1A*, **G** *COX IV*, **H** *COX II*, **I** *CYTB*, **J** *ND4*, following 24 and 48 h of lactate exposition, analyzed by Real-time PCR. The calculated value of 2^−*ΔΔ*Ct^ in untreated controls is 1. **K** Cytoplasm Mitotracker Intensity. Data are expressed as mean ± SD of at least four independent experiments. (*P < 0.05; **P < 0.005; ***P < 0.001; ****P < 0.0001). Scale bars in **K** 10 μm

We firstly evaluated the effect of lactate on microglia cells viability. As shown in Fig. 1A, lactate 20 mM (48 h) resulted in a significant increase in MTT turnover (135.67% ± 10.03%) as compared to untreated control cells (100 ± 3.7%, Fig. 1A). Therefore, we used this concentration for subsequent experiments. We then evaluated the effect of lactate on MCTs expression and in mitochondrial metabolism by evaluating mitochondrial biogenesis and oxidative phosphorylation in microglia cells. Our results showed a significant increase in relative mRNA levels of *SLC16A1(MCT1)* (6.97 ± 0.55) and *SLC16A3 (MCT4)* (2.57 ± 0.3) when exposed to 48 h as compared to untreated control cells (Fig. 1B,C). Because oxidative cancer cells expressing MCT1 are capable of taking up lactate secreted by glycolytic cancer cells, we analyzed mRNA expression of a panel of genes involved in mitochondrial metabolism. Our results showed that lactate treatment induced a significant change in the expression of genes involved in mitochondrial biogenesis and oxidative phosphorylation. As shown in Fig. 1D–J, following lactate exposure, microglia cells showed a significant increase in relative mRNA levels of *PGC1α* (48 h: 6.97 ± 0.23, Fig. 1D) and *TFAM* (24 h: 1.2 ± 0.05, Fig. 1E), coupled with an overall increase in *COX IV* (24 h: 1.23 ± 0.07, Fig. 1G), *COX II* (24 h: 1.40 ± 0.18, Fig. 1H), *CYTB* (24 h: 1.45 ± 0.13, Fig. 1I) and *ND4* (24 h: 1.53 ± 0.11, Fig. 1J) compared to untreated control cells. We also performed analysis of mitotracker fluorescence intensity on control versus lactate treated cells, showing that lactate was able to significantly increase cytoplasm mitotracker intensity 18 h following treatment (Fig. 1K).

### Lactate promotes microglia M2 polarization through the expression of IGFBP6

To further confirm whether lactate serves as an oncometabolite driving anti-inflammatory polarization of microglia in TME and promoting tumor progression, we assessed the effect of lactate exposure on the expression of pro- and anti-inflammatory markers. Firstly, we evaluated the expression of *ARG1, CD206, CD163, TGFß, IL6 and TNF* genes. Our data suggested that after 24 and 48 h incubation, lactate treatment showed a significant increase in relative mRNA expression levels of M2-like phenotype markers *ARG1* (24 h: 2.70 ± 0.23, 48 h: 2.50 ± 0.14, Fig. S1A), *CD206* (24 h: 2.37 ± 0.09, 48 h: 4.25 ± 0.12, Fig. S1B), *CD163* (48 h: 4.47 ± 0.34, Fig. S1C), *TGFß* (24 h: 2.67 ± 0.27, 48 h: 2.68 ± 0.23, Fig. S1D) and *IL6* (24 h: 1.77 ± 0.18, 48 h: 1.55 ± 0.08 Fig. S1E). Consistently, these results were confirmed by immunocytochemistry analysis, showing that lactate treated cells (72 h) showed lower levels of iNOS (M1-like phenotype marker) and higher levels of ARG1 (M2-like phenotype marker) compared to their untreated control cells (Fig. S1G-H).

**Fig. 2:**
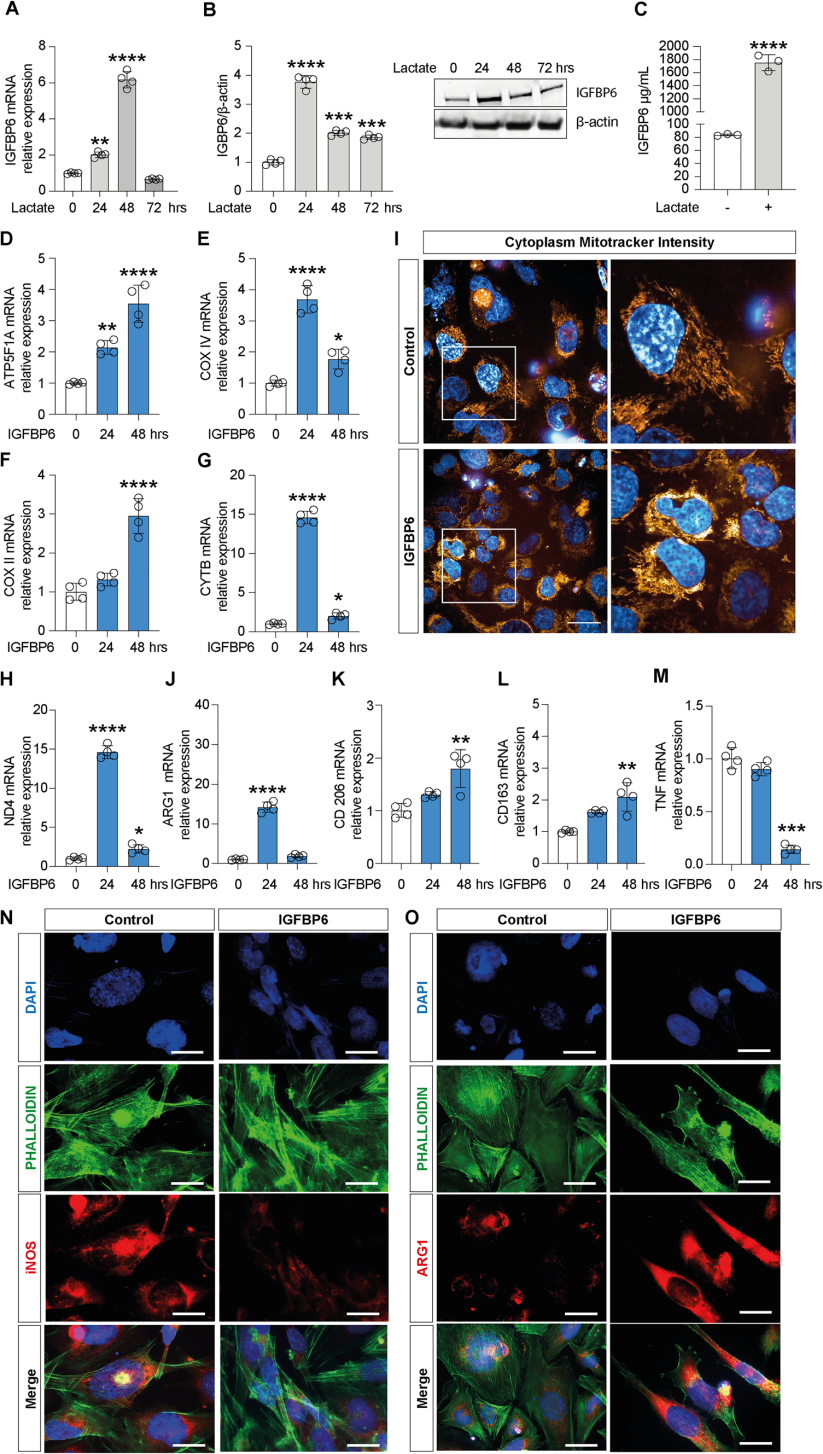
Lactate induces IGFBP6 expression which promotes M2-like phenotype polarization of microglia. Effect of lactate on IGFBP6 **A** mRNA expression levels, **B** protein expression and **C** production in microglia cells. Evaluation of relative mRNA expression levels of **D** *ATP5F1A*, **E** *COX IV*, **F** *COX II*, **G** *CyTB*, **H** *ND4*. **I** Cytoplasm Mitotracker Intensity. Evaluation of relative mRNA expression levels of **J** *Arg1,* **K** *CD 206*, **L** *CD 163* and **M** *TNF*, analyzed by real-time PCR. The calculated value of 2^−*ΔΔ*Ct^ in untreated controls is 1. Immunocytochemistry analysis of **N** iNOS and **O** Arg1, following 72 h of IGFBP6 treatment. Data are expressed as mean ± SD of at least four independent experiments. (*P < 0.05; **P < 0.005; ***P < 0.001; ****P < 0.0001). Scale bars in (**I, N** and **O**) 10 μm

Given the evidence on cellular modulation exerted by increased extracellular levels of lactate, we sought to link molecular mechanisms underlying these phenomena with the expression of IGFBP6. Therefore, we first analyzed the effect of lactate on the expression of IGFBP6. Our results (Fig. 2A-B) showed that treatment with lactate in microglia cells resulted in a significant increase in both relative mRNA (24 h: 2.02 ± 0.14, Fig. 2A) and protein (24 h: 3.77 ± 0.21, Fig. 2B) expression levels compared with untreated control cells. These data were confirmed by the ELISA test performed on cell culture supernatants (1752.6 ug ± 123.5 lactate versus 83.6 ug ± 1.29 control, Fig. 2C). Furthermore, we also treated microglia cells with the recombinant protein IGFBP6 (400 ng/mL) for 24 and 48 h and evaluated whether IGFBP6 was also able to promote oxidative metabolism and M2 polarization. Consistently, we showed that, similarly to lactate, IGFBP6 was also able to induce a significant increase in relative mRNA expression levels of genes involved in oxidative phosphorylation pathway, i.e. *ATP synthase F1 subunit alpha* (*ATP5F1A)* (24 h: 2.14 ± 0.21, Fig. 2D), *COX IV* (24 h: 3.69 ± 0.44, Fig. 2E), *COX II* (24 h: 1.32 ± 0.15, Fig. 2F), *CYTB* (24 h: 14.59 ± 0.8, Fig. 2G) and *ND4* (24 h: 14.21 ± 1.2, Fig. 2H), confirming that IGFBP6 promotes oxidative metabolism in microglia cells. These data were further confirmed by mitotracker fluorescence intensity analysis, showing that IGFBP6 treated cells showed significantly increase in cytoplasm mitotracker intensity 18 h post-treatment compared to control cells (Fig. 2M). Then, we evaluated the effect on M1-like and M2-like phenotype markers, as shown in Fig. 2J–O. In this regard, our results showed an increase in *ARG1* (24 h: 14.21 ± 1.2, 48 h: 1.87 ± 0.36, Fig. 2J), *CD206* (48 h: 1.80 ± 0.36, Fig. 2K), *CD163* (48 h: 2.09 ± 0.45, Fig. 2L) and a decrease in *TNF* (48 h: 0.14 ± 0.03, Fig. 2M) mRNA expression levels in IGFBP6 treated microglia cells compared to untreated control cells. Furthermore, we confirmed these results by immunocytochemistry analysis, which showed a reduction in iNOS (Fig. 2N) expression and an increase in ARG1 (Fig. 2O) expression following 72 h of IGFBP6 treatment compared to untreated cells.

### IGFBP6 enhances migration and colony formation capacity in glioblastoma cells

**Fig. 3:**
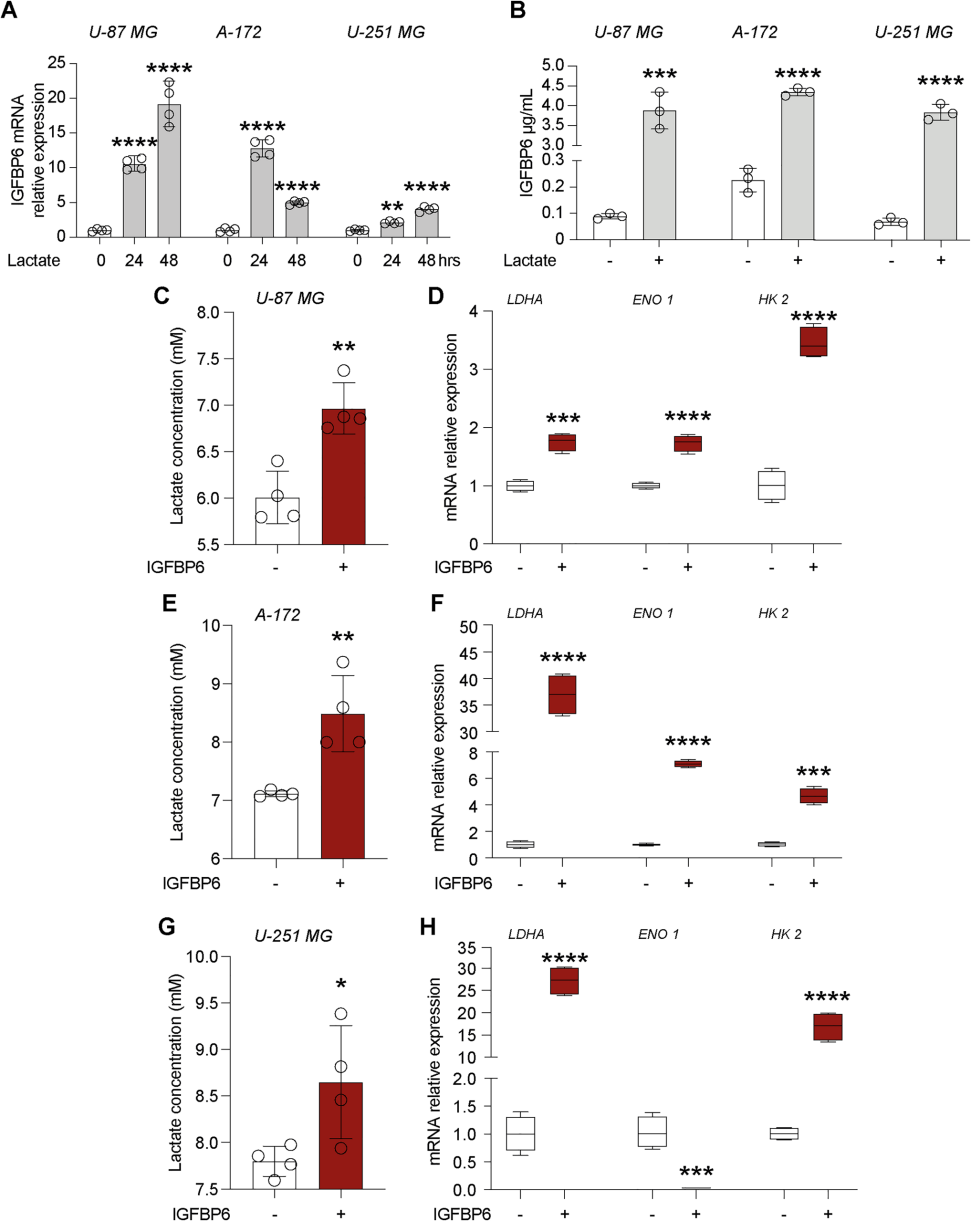
Lactate and IGFBP6 crosstalk. Effect of lactate on IGFBP6 **A** mRNA expression levels and **B** production in Glioblastoma cells. Effect of IGFBP6 treatment on lactate production in (**C**) U-87 MG, **E** A-172 and **G** U-251 MG cells. Evaluation of mRNA expression levels of LDHA, ENO1 and HK2 in **D** U-87 MG, **F** A-172 and **H** U-251 MG cells

**Fig. 4:**
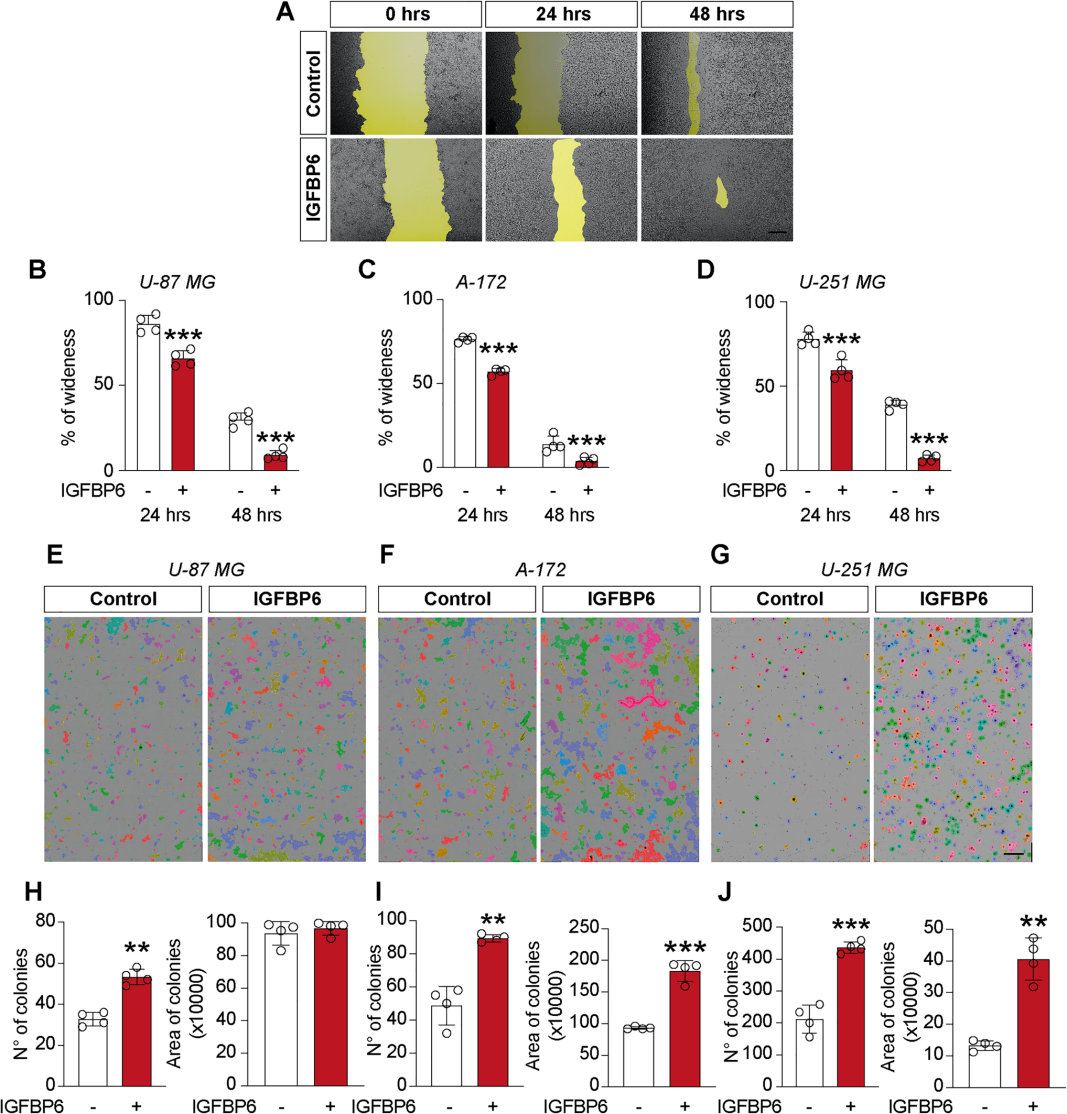
IGFBP6 enhances migratory and colony formation capacity in glioblastoma cells. Effect of IGFBP6 treatment on migratory capacity **A**–**D** and colony formation capacity **G**–**H** in Glioblastoma cells. Data are expressed as mean ± SD of at least four independent experiments. (*P < 0.05; **P < 0.005; ***P < 0.001; ****P < 0.0001). Scale bar in (**A**) 100 μm and scale bar in (**E**, **F** and **G**) 1 mm

Microglia plays an important role in the microenvironment that supports Glioblastoma progression. Since lactate promotes tumor proliferation and migration in GBM cells, we evaluated the effect of lactate on IGFBP6 expression and the effect of IGFBP6 exposure in three human GBM lines (i.e. U-87 MG, A-172 and U-251 MG). Interestingly, our results showed that in three GBM cell lines (U-87 MG, A-172 and U-251 MG), lactate induced a significant increase in relative IGFBP6 mRNA expression levels following 24 h (U-87 MG: 10.48 ± 0.8; A-172: 12.79 ± 1.2; U-251 MG: 2.11 ± 0.16, Fig. 3A) and 48 h (U-87 MG: 19.16 ± 2.9; A-172: 4.96 ± 0.3; U-251 MG: 4.02± 0.3, Fig. 3A) of treatment compared to their untreated control cells. Similarly, we evaluated IGFBP6 production in cell culture supernatant following 24 h of lactate treatment, showing that IGFBP6 production was significantly increased in the supernatant of lactate treated U-87 MG (3.87 ± 0.46 µg/mL, Fig. 3B) cells compared to control cells (0.089 ± 0.01 µg/mL, Fig. 3B). The same results were observed in A-172 (4.34 ± 0.09 µg/mL lactate versus 0.23 ± 0.04 µg/mL, Fig. 3B) and U-251 MG (3.54 ± 0.25 µg/mL lactate versus 0.07 ± 0.01 µg/mL control, Fig. 3B) cells. Therefore, in order to demonstrate a possible link between lactate and IGFBP6, we also evaluated lactate production in supernatants of IGFBP6-treated GBM cells and whether IGFBP6 exposition affects expression of metabolic enzymes and cell migration on GBM cell lines. Interestingly, our results showed a significant increase in lactate production in supernatants of IGFBP6-treated cells, in all three tested cell lines U-87 MG (6.97 ± 0.3 mM lactate versus 6.01 ± 0.3 mM control, Fig. 3C), A-172 (8.5 ± 0.7 mM lactate versus 7.1 ± 0.05 mM control, Fig. 3E) and U-251 MG (8.65 ± 0.6 mM lactate versus 7.8 ± 0.16 mM control, Fig. 3G). Furthermore, exposure to IGFBP6 for 24 h in GBM cells resulted in a significant increase in *lactate dehydrogenase A* (*LDHA)* mRNA levels in U-87 MG (1.75 ± 0.15, Fig. 3D), A-172 (36.9 ± 3.9, Fig. 3F) and U-251 MG (27.2 ± 3.2, Fig. 3H) compared to their control cells, a significant increase in *hexokinase 2* (*HK2)* mRNA expression levels in all three cell lines (Fig. 3D–H), and a significant increase in *Enolase 1* (*ENO1)* mRNA expression levels only in U-87 MG and A-172 cells but not in U-251 MG (Fig. 3D–H). Consistently, we observed a reduced percentage of wound wideness at 48 h in U-87 MG (9.08 ± 2.73% IGFBP6 versus 29.9 ± 3.9% control, Fig. 4B), A-172 (3.86 ± 2.3% IGFBP6 versus 13.84 ± 4.9% control, Fig. 4A–C) and U-251 MG (7.25 ± 2.01% IGFBP6 versus 39.97 ± 2.5% control, Fig. 3D) cells. We also confirmed the effects of IGFBP6 on colony formation capacity, finding a significantly increased in colonies number (53.25 ± 3.7 IGFBP6 versus 32.75 ± 3.31 control, Fig. 4E–H) but not in the growth area of each colony in U-87 MG cells (Fig. 4E–H). A-172 cells exhibited a significant increase in both the number of colonies (89.25 ± 2.21 IGFBP6 versus 48.75 ± 11.6 control, Fig. 4F–I) and the colonies area (1.83 ± 0.16 mm^2^ IGFBP6 versus 0.93 ± 0.01 mm^2^ control, Fig. 4F–I). Similar results were obtained in U-251 MG cells, showing an increase in colonies number (436.5 ± 17.8 IGFBP6 versus 212 ± 44.2 control, Fig. 4G–J) and in colonies area (0.4 ± 0.06 mm^2^ IGFBP6 versus 0.13 ± 0.01 mm^2^ control, Fig. 4G–J).

### IGFBP6 increases sonic hedgehog signalling pathway activation in GBM and microglia

**Fig. 5:**
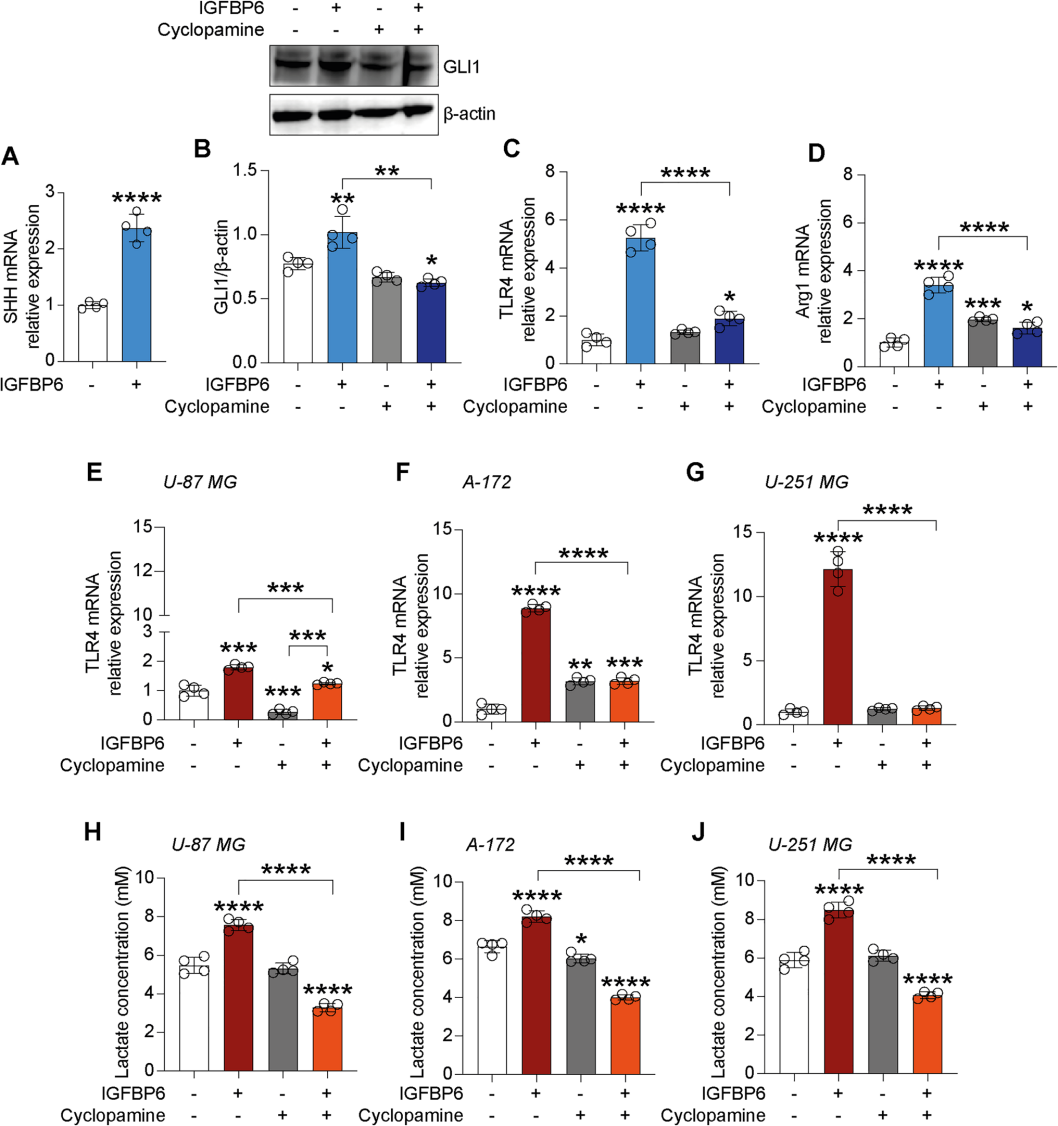
IGFBP6 increases sonic hedgehog signalling pathway activation in GBM and microglia.Evaluation of mRNA expression levels of SHH in microglia cells treated with IGFBP6 **A**, and GLI1 protein expression after IGFBP6 and cyclopamine treatment **B**. Evaluation of mRNA expression levels of TLR4 **C** and ARG1 **D** after IGFBP6 and cyclopamine treatment in microglia cells. Evaluation of mRNA expression levels of TLR4 after IGFBP6 and cyclopamine treatment in U-87 MG **E**, A-172 **F** and U-251 MG **G** cells. Effect of IGFBP6 and cyclopamine treatment on lactate production in U-87 MG **H**, A-172 **I** and U-251 MG **J** cells. Data are expressed as mean ± SD of at least four independent experiments. (*P < 0.05; **P < 0.005; ***P < 0.001; ****P < 0.0001)

In an attempt to link the effects of IGFBP6 with key inducers and regulators of cell fate and proliferation, we investigated whether IGFBP6 stimulation was coupled to SHH deregulation again. Surprisingly, IGFBP6 stimulation was associated with significant increase in *SHH* mRNA expression level (2.37 ± 0.25 IGFBP6 versus 1.00 ± 0.06 control, Fig. 5A) and increased expression of the GLI zinc finger 1 family protein (GLI1) (1.02 ± 0.12 IGFBP6 versus 0.77 ± 0.05 control, Fig. 5B), indicating that IGFBP6 stimulation was able to induce the canonical SHH signaling pathway on HMC3 microglial cells, and that this effect was reversed by co-treatment with cyclopamine (i.e. inhibitor of the SHH pathway) (0.63 ± 0.0. IGFBP6/cyclopamine versus 1.02 ± 0.12 IGFBP6, Fig. 5B), but this did not happen in glioblastoma cells (Figure S2). Similarly, we analyzed *TLR4* mRNA expression levels after exposure to IGFBP6 and/or cyclopamine, showing that IGFBP6 was capable of inducing significant increased expression of *TLR4* mRNA both in microglia (5.27 ± 0.55 IGFBP6 versus 1.00 ± 0.25 control, Fig. 5C) and GBM cells (U-87 MG: 1.80 ± 0.08 IGFBP6 versus 1.00 ± 0.18 control, Fig. 5E; A-172: 8.89 ± 0.30 IGFBP6 versus 1.00 ± 0.38 control, Fig. 5F; U-251 MG: 12.15 ± 1.34 IGFBP6 versus 1.00 ± 0.21 control, Fig. 5G) and that this effect was reversed by IGFBP6/cyclopamine co-treatment (HMC3: 1.89 ± 0.31, Fig. 5C; U-87 MG: 1.25 ± 0.04, Fig. 5E; A-172: 3.19 ± 0.27, Fig. 5F; U-251 MG: 1.31 ± 0.18, Fig. 5G). Interestingly, IGFBP6/cyclopamine co-treatment in HMC3 cells also resulted in a significant reduction in ARG1 mRNA expression levels (1.61 ± 0.25 IGFBP6/cyclopamine versus 3.41 ± 0.34 IGFBP6, Fig. 5D).

Furthermore, in order to verify whether the IGFBP6/SHH/TLR4 axis was involved in lactate production, we also analyzed, in GBM cells, the concentration of lactate produced after IGFBP6 and/or cyclopamine exposure, showing that in all three glioblastoma cell lines IGFBP6 was able to induce a significant increase in lactate production and that this effect was significantly reversed by inhibition of SHH signaling by cyclopamine (U-87 MG: 5.47 ± 0.43 mM control, 7.58 ± 0.29 mM IGFBP6, 5.32 ± 0.29 mM cyclopamine, 3.29 ± 0.22 mM IGFBP6/cyclopamine, Fig. 5H; A-172: 6.65 ± 0.32 mM control, 8.20 ± 0.30 mM IGFBP6, 6.03 ± 0.23 mM cyclopamine, 4.00 ± 0.14 mM IGFBP6/cyclopamine, Fig. 5I; U-251 MG: 5.90 ± 0.39 mM control, 8.49 ± 0.41 mM IGFBP6, 6.11 ± 0.30 mM cyclopamine, 4.07 ± 0.16 mM IGFBP6/cyclopamine, Fig. 5J).

### GBM derived IGFBP6 promotes microglia M2 polarization

**Fig. 6:**
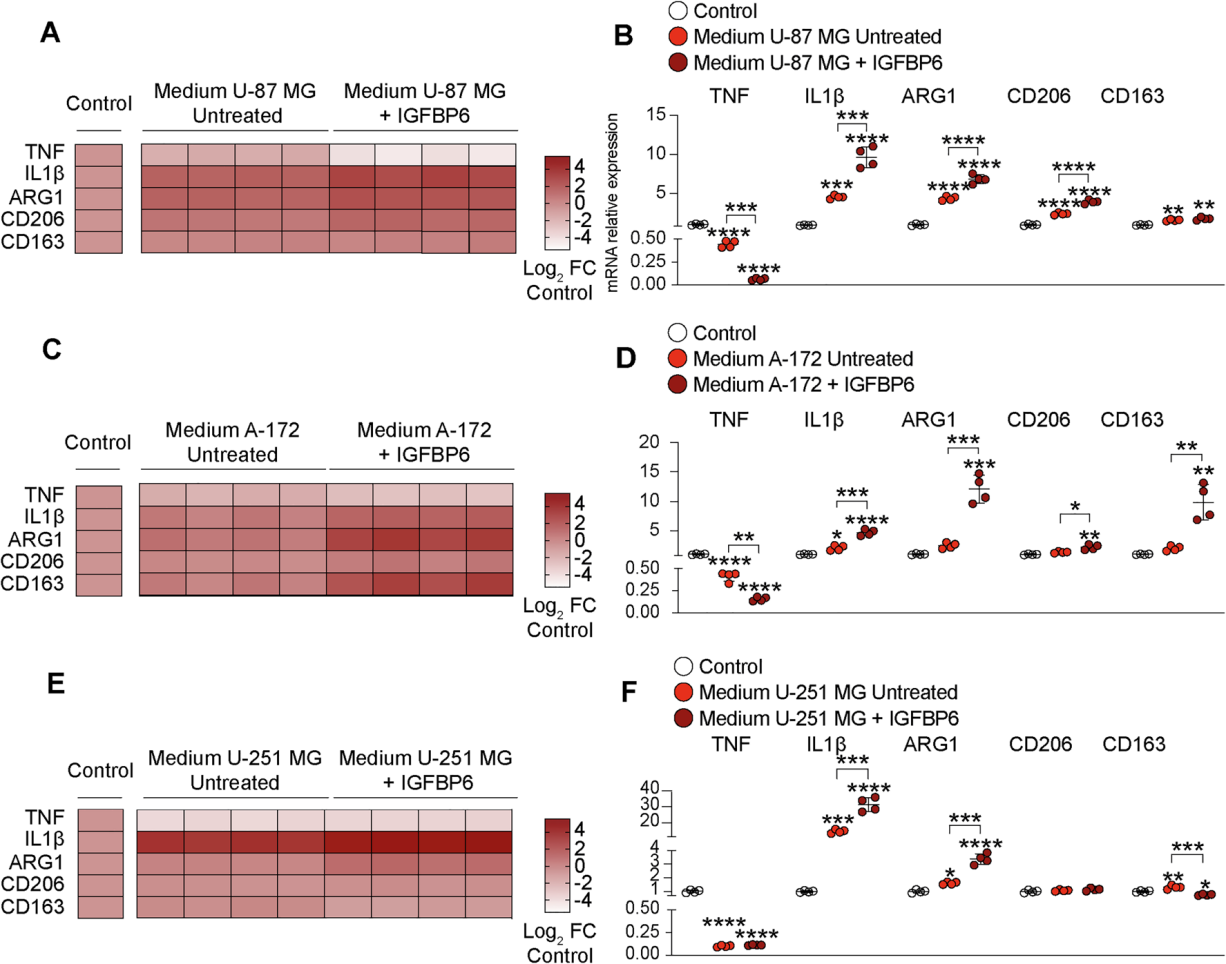
IGFBP6 produced by glioblastoma cells promote microglia M2-like phenotype polarization.Evaluation of mRNA expression levels of TNF, IL1β, ARG1, CD206 and CD163 in microglia cells treated with conditioned medium of **A-B** U-87 MG, **C-D** A-172, **E-F** U-251 MG pre-treated with IGFBP6. Data are expressed as mean ± SD of at least four independent experiments. (*P < 0.05; **P < 0.005; ***P < 0.001; ****P < 0.0001)

Microglia exposed to the conditioned medium derived from U-87 MG cells pre-treated with IGFBP6, showed a significant decrease in relative mRNA expression levels of *TNF* and a significant increase in relative mRNA expression levels of *IL1b*, *ARG1*, *CD206* and *CD163* compared to both control cells and not pre-treated cells with IGFBP6 (Fig. 6A–B, Table S1). Consistently, our results showed a similar effect in microglia exposed to the conditioned medium derived from of A-172 cells pre-treated with IGFBP6, as compared to control and untreated cells exposed to A-172 medium (Fig. 6C–D, Table S1). Interestingly, the conditioned medium from U-251 MG cells pre-treated with IGFBP6 had no significant effect on *TNF* and *CD206* relative mRNA expression levels compared to their controls (Fig. 6E–F, Table S1).

### Lactate induces changes in *igfbp6b* expression in a zebrafish brain tumor model

**Fig. 7:**
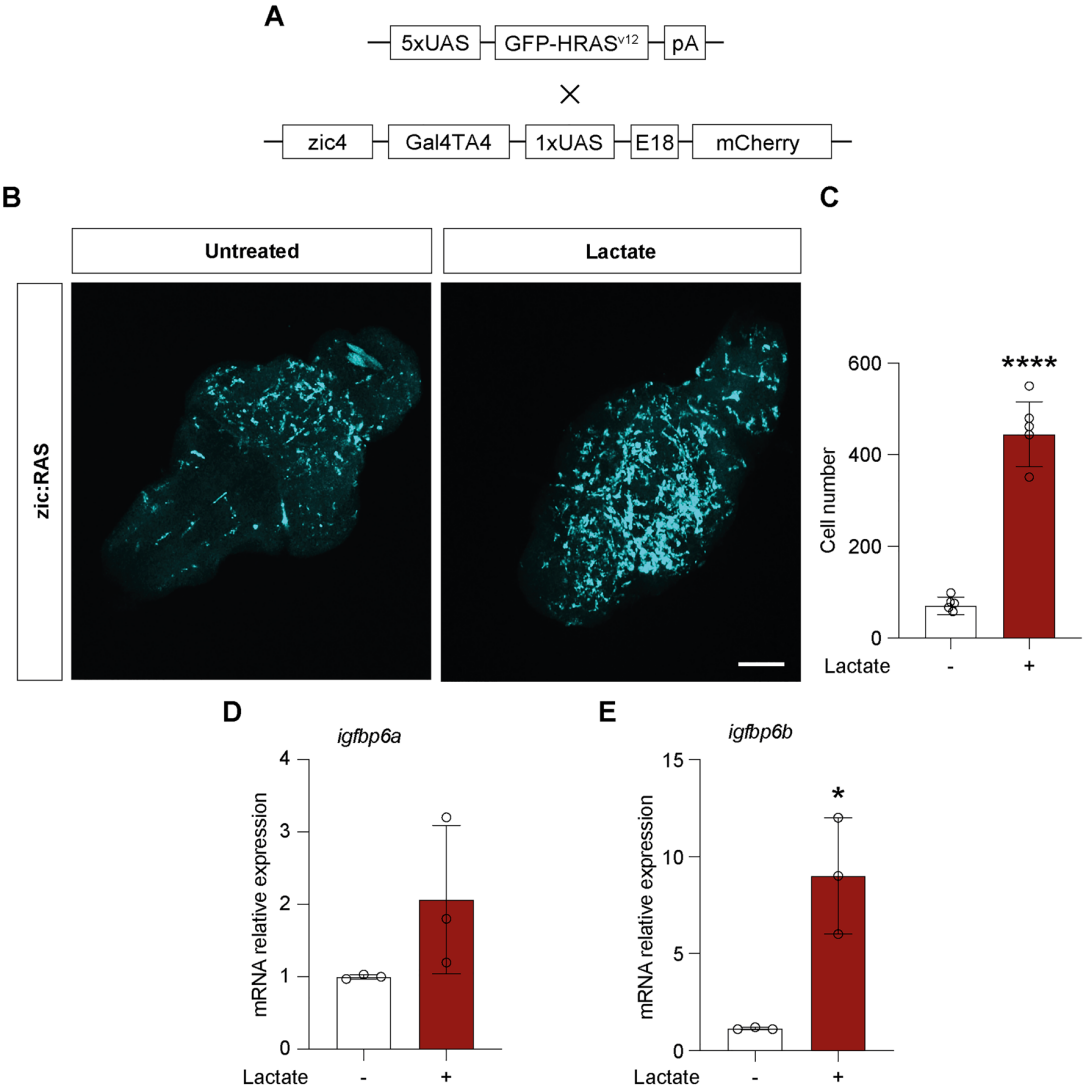
Lactate induces changes in microglia and *igfbp6b* expression in a zebrafish brain tumor model **A** Schematic representation of the genetic components of the zebrafish GBM model; **B** whole mount immunofluorescence of microglia (cyano) in zic:Ras larval brains treated as indicated; **C** count of microglia cells in whole zic:RAS control brains and treated with 20 mM lactate, data represent the means of counts performed on 5 biological replicates for each condition; d) gene expression analysis of *igfbp6a* and *igfbp6b* in 5dpf zic:Ras control larvae and treated with 20 mM lactate. (****P < 0.0001; *P < 0.01). Scale bar in **B** 40 μm

To investigate the effects of lactate accumulation on GBM and microglia cells in vivo, we used a zebrafish model of Glioblastoma [37] (Fig. 7A). The levels of expression of *igfpb6a* and *igfbp6b* in response to lactate have been analyzed through qPCR on total RNA extracted from larvae developing GBM, incubated with lactate as described above. Q-PCR analysis did not show significant changes in *igfpb6a* expression levels (Fig. 7B) in zic:RAS larvae treated with lactate, in comparison to not-treated zic:RAS larvae. On the contrary, the expression of *igfbp6b* appeared to be significantly increased upon lactate treatment (Fig. 7C). We also analyzed the effects of lactate on microglia number by immunostaining using the microglia marker L-plastin (Fig. 7D). Incubation of developing larvae from 2 to 5 dpf with 20 mM leads to a significant increase in the number of cells expressing L-plastin in brains of zic:RAS larvae, compared to non-treated zic:RAS brains (Fig. 7E).

### IGFBP6 was modulated in GBM patients

**Fig. 8:**
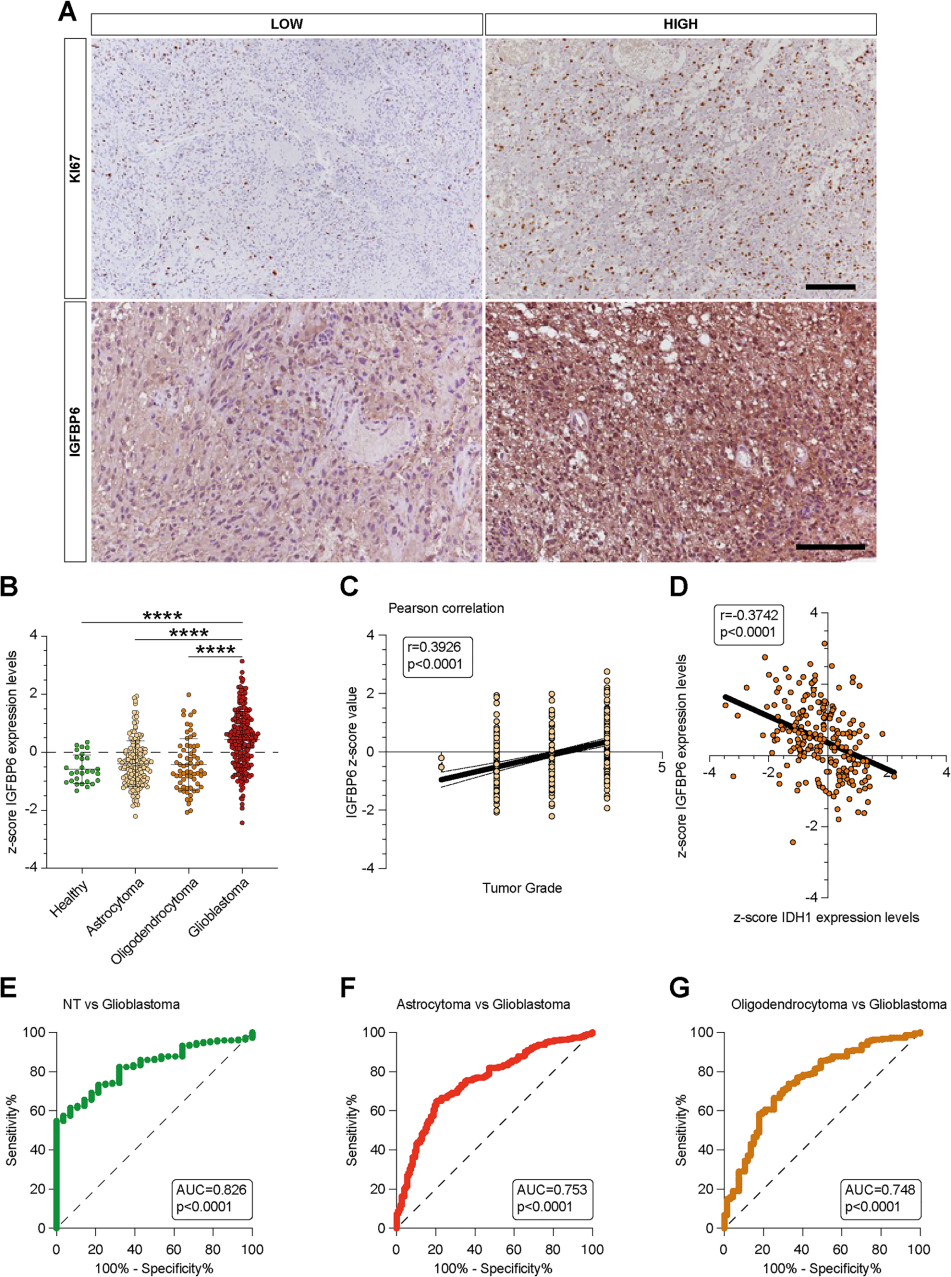
IGFBP6 was modulated in GBM patients.Analysis of IGFBP6 gene expression in brain biopsies of patients with astrocytoma, oligodendrocytoma, glioblastoma, and healthy subjects. **b** Pearson correlation analysis between IGFBP6 expression levels and tumor grade of brain biopsies obtained from patients affected by main brain tumors. **c** Pearson's correlation between IGFBP6 and IDH1 expression levels in brain biopsies of patients with glioblastoma. **d** Receiver operating characteristic (ROC) analysis between IGFBP6 brain expression levels in healthy subjects vs glioblastoma patients, between glioblastoma patients vs astrocytoma patients **e**, and vs oligodendrocytoma **f**. Data are expressed as mean ± SD of at least four independent experiments. (*P < 0.05; **P < 0.005; ***P < 0.001; ****P < 0.0001). Scale bar in **A** 50 μm

Across the whole cohort of GBM patients, the immunohistochemical expression of IGFBP6 was high (IRS ≥ 6) in 5 cases (50%) and low (IRS < 6) in the remaining 5 cases (50%). Interestingly, all cases (5 out of 5) that showed high immunohistochemical expression of IGFBP6, also exhibited MIB-1 levels > 50% (Fig. 8); conversely, the remaining cases (5 out of 5) with low IGFBP6 immunoexpression had low MIB-1 proliferative rates (< 50%). A positive correlation was found between IGFBP6 immunohistochemical expression and MIB-1 proliferative rate in all GBM cases examined.

### IGFBP6 gene expression analysis in human glioma

The IGFBP6 gene expression analysis obtained from the GSE108474 dataset showed that there were significant differences when the expression levels obtained from brain biopsies of glioblastoma patients were compared to the other brain tumors stages. (Fig. 8). Specifically, patients with glioblastoma expressed significantly higher levels of the IGFBP6 messenger in the brain than patients with oligodendroglioma (*P* < 0.0001), astrocytoma (*P* < 0.0001), or healthy subjects (*P* < 0.0001) (Fig. 5a). This finding was confirmed by the significantly positive correlation between IGFBP6 expression levels and tumor grade (*r* = 0.3926; *P* < 0.0001) (Fig. 8B). According to these results, we investigated the prognostic potential of IGFBP6 expression in the progression of main brain tumors. Currently, the expression analysis of Isocitrate Dehydrogenase (NADP (+)) 1 (IDH1) and the identification of its main mutations (e.g. R132H) are used for glioma diagnosis and prognosis. By carrying out a Pearson correlation analysis between IGFBP6 and IDH1 brain tumor expression levels, we highlighted that in glioblastoma patients, the expression levels of the two genes were significantly closely inversely correlated (*r* = -0.3743, *P* < 0.0001) (Fig. 8C). Furthermore, in order to evaluate the potential diagnostic ability of IGFBP6 gene expression to discriminate against the brain tumors stages, we performed a Receiver operating characteristic (ROC) analysis. We confirmed the diagnostic ability of IGFBP6 to discriminate the glioblastoma patients from healthy subjects (AUC = 0.826, *P* < 0.0001) (Fig. 8D) or from the patients affected to astrocytoma (AUC = 0.753, *P* < 0.0001) (Fig. 8E) or oligodendrocytoma (AUC = 0.748, *P* < 0.0001) (Fig. 8F).

## Discussion

The extensive production of acidic metabolites and the enhanced acid export to the extracellular space results in a significant acidification of TME, thus promoting the formation of an acid-resistant tumor cell population with increased invasiveness and metastatic potential [7]. Lactic acidosis was reported in the TME of several tumors including GBM triggering a series of biochemical mechanisms modifying cell metabolism and signaling; furthermore, a variety of oncogenic and environmental factors alter tumor metabolism to meet the distinct cellular biosynthetic and bioenergetic needs present during oncogenesis [34]. Among the most interesting biological processes reshaping cell metabolism, those that recap developmental processes, such as the one involving IGFBP6, hold great potential to understand tumor-related biological processes such as cell proliferation, migration, senescence and changes in metabolism [18]. For this purpose, we aimed at evaluating the potential crosstalk between lactate and IGFBP6 in microglial cells and the impact of such interaction on TME and GBM progression. Our hypothesis was supported also by several studies showing that in the early phases of the disease, GAMs are abundant in the tumor mass and infiltrate TME contributing to tumorigenesis [35].

Firstly, we showed that exposing microglia cells to lactate results in a significant increase in MCT1 gene and protein expression, responsible for the intracellular influx of lactate and involved in lactate shuttling to provide energetic support. Consistent with our data, Moreira et al. demonstrated that microglia express MCT1 and MCT2 responsible for lactate and ketones uptake [36]. Our data also showed that lactate also induces a significant up-regulation of genes involved in oxidative phosphorylation. Interestingly, MCT1 has a high affinity for lactate and is preferentially expressed in oxidative cells that take up lactate [37]. In oxidative cells, lactate reacts intracellularly with NAD^+^ to yield pyruvate, NADH and H + (the LDHB reaction), and both pyruvate and NADH can fuel the TCA cycle and OXPHOS in mitochondria, which depends on the malate–aspartate shuttle for the mitochondrial import of NADH [37]. Interestingly, Gasior et al. showed that a ketogenic diet correlates with a suppression of microglia activation [38]. Therefore, we verified the ability of lactate to promote M2 polarization (immunosuppressive phenotype) of microglia, showing both by gene expression and immunocytochemical analysis an increase in the expression of M2 markers (i.e., ARG1, CD 206, CD 163) and a decrease in M1-like phenotype markers (iNOS) in treated cells as compared to untreated cells, suggesting that lactate was able to induce modification of microglia metabolic functions. To this regard, recent data confirmed the link between metabolism and microglia polarization, similar to that of traditional macrophages. Our data are consistent with the study by Xianmin Mu et al., demonstrating that tumor-derived lactate induced M2 macrophage polarization [39], via ERK/STAT3 signaling thus facilitating angiogenesis, cancer cell migration, and invasion and that lactate correlated with cell re-education in the TME [40]. Moreover, increased glycolysis is observed in M1-like microglia, which is dependent on the increase in hexokinase and lactate dehydrogenase activity, and high expression of GLUT1 [41]. Similarly, Colegio et al. found that tumor-associated macrophage (TAM) polarization is dependent on tumor-derived lactic acid, and the mechanism is mediated by hypoxia-inducible factor 1α (HIF1α) [40]. However, the pathways driven by lactate eliciting M2-like functional polarization of TAMs are still not fully elucidated. Indeed, a number of signaling pathways are involved in M2-like macrophage polarization, including ERK1/2, STAT3, HIF1α, STAT6 [39]. Furthermore, several studies also showed that lactic acid was sufficient to induce macrophage polarization via pH acidification of TME [39], even though the cellular mechanisms and molecular pathways involved in such a modulation remain elusive.

Thus, given the evidence on cellular modulation exerted by increased extracellular levels of lactate, we sought to link metabolic reshaping with IGFBP6 and found that lactate modulates microglia anti-inflammatory polarization and remodels TME in GBM through IGFBP6.

Interestingly, we found that microglia exposed to lactate showed a significant increase in both gene and protein expression of IGFBP6. These results were confirmed by measuring the levels of IGFBP6 in the supernatant of lactate-treated cells, which showed a significant increase in the production of IGFBP6 compared to control cells and its release to the extracellular milieu. These data suggest that lactate influences IGFBP6 signaling. This is consistent with the well-known IGF system role and its interaction with cell metabolism. The regulation of IGF pathway plays a key role in a number of common disease processes, including cancer [42]. In our previous work, we investigated the involvement of IGFBP6, sonic hedgehog (SHH) and Toll-like receptor 4 (TLR4) axis in alterations of TEM [21], and we found that both IGFBP6 and purmorphamine, an activator of SHH, were able to induce differentiation of mesenchymal stromal cells and that TLR4 signaling was activated after exposure to IGFBP6 and purmorphamine and restored by exposure to cyclopamine, an inhibitor of the SHH pathway, confirming that SHH is involved in activation of TLR4 and alterations of the microenvironment, suggesting that the IGFBP6/SHH/TLR4 axis is implicated in alterations of the tumor microenvironment. Herein, we observed that such a phenomenon was recapitulated in microglial cells exposed to IGFBP6 and that such an effect was coupled with an increased SHH mRNA level. Moreover, IGFBP6 effects on SHH signalling pathway were reverted by co-treatment with effector antagonist cyclopamine.

Surprisingly, IGFBP6 treatment was also able to upregulate oxidative phosphorylation and to induce an immunosuppressive phenotype in microglia cells. These data are consistent with the study of Chesik D. et al., in which they examined the expression of IGFBPs (from 1 to -6) in primary rat microglia cultures under basal conditions and after stimulation with LPS, a well-established activator of microglia, demonstrating a significant down-regulation of IGFBP-4, -5 and -6 [43].

In recent years, IGFBP6 has been shown to inhibit IGF-II and to modulate nuclear transcription of genes involved in differentiation and survival, leading to increase cell migration [44]. Indeed, GBM cells exposed to IGFBP6 significantly increased LDHA mRNA expression, thus confirming the existence of IGFBP6 to lactate axis that increases cell proliferation and colony-forming capacity. This finding was confirmed in our cohort of GBM biopsies. In particular, we assessed the relationship between the immunohistochemical expression of IGFBP6 and MIB-1, a widely used marker of the tumor proliferative activity, and found a positive correlation between these two markers. In our series, while GBM cases that showed MIB-1 levels > 50%, exhibited a strong and diffuse positivity for anti-IGFB6 antibody, those with low MIB-1 proliferative index (< 50%) were weakly and patchily stained with IGFB6. Based on these findings, it can be hypothesized that IGFBP6 may be used in the future as easily detectable prognostic marker of GBM, predictor of increased tumor aggressiveness.

Besides the role of microglia-derived IGFBP6 on GBM cells, we also observed that conditioned medium from IGFBP6 treated GBM cells was able to modulate microglia polarization, inducing an M2-like phenotype. Consistent with these data, Li et al. showed that in GBM, microglial cells showed to have a pro-tumor phenotype that is associated with the M2-like phenotype of macrophages due to its expression of specific factors, such as ILs, transforming growth factor beta 1 (TGF-β1), monocyte chemoattractant protein (MCP-1), and prostaglandin E2 (PGE-2) [45]. At the same time, the glial cells from TME also release factors that support GBM growth. Furthermore, other studies of Lisi et al. suggested that in the case of GBM, glioma cells are able to suppress the microglial M1-like phenotype and induce an anti-inflammatory phenotype through the abovementioned cytokines and chemokines, which in turn induce microglial cells to release different factors that will stimulate tumor growth [46]. Moreover, the presence of microglial M2-like phenotype has been associated with the aggressiveness and poor prognosis in GBM patients [4]. These results are consistent with hallmarks of microglial activation seen in cases of disease and histopathological analysis of GBM tumors. Interestingly, several studies have shown that IGFBP-3, known to regulate cell proliferation, was also increased in GBM-microglia crosstalk, though its role in cancer progression remains to be fully understood [47].

Our results were further confirmed by the analysis of human GSE108474 dataset showing that IGFBP6 was significantly modulated during the progression of the disease. In particular, significant expression changes were highlighted with the increase in the degree of malignancy. Furthermore, our results showed that IGFBP6 discriminated patients with glioblastoma versus those with astrocytoma and oligodendroglioma. These data are consistent with previous studies showing that IGFBP6 is a prognostic biomarker in human glioblastoma [48]. Interestingly, there exists an inverse correlation between IGFBP6 and IDH1 expression in glioblastoma patients. Currently, no data is available demonstrating the correlation between IGFBP6 and IDH1 and future studies are now warranted in order to further evaluate such correlation.

In conclusion, our results demonstrate that IGFBP6 modulates polarization of microglia and that its expression is regulated by lactate production in GBM cells suggesting the existence of a lactate to IGFBP6 crosstalk between microglial cells and GBM and this relationship modulates TME, which could affect tumor progression and resistance to therapy and that the complex network of interaction between microglial cells and GBM may represent a potential therapeutic target to overcome tumor malignancy.

## Supplementary Information

Below is the link to the electronic supplementary material.

Supplementary file1 (DOCX 2161 KB)

Supplementary file2 (DOCX 3226 KB)
