# Supplementary material for: Lactate modulates microglia polarization via IGFBP6 expression and remodels tumor microenvironment in glioblastoma
Source: Cancer Immunol Immunother. 2022 Jun 3;72(1):1–20. doi: 10.1007/s00262-022-03215-3 (PMC9813126; doi:10.1007/s00262-022-03215-3)

**Figure S1. Lactate promotes M2-like phenotype polarization of microglia.** Evaluation of relative mRNA expression levels of (**A**) ARG1, (**B**) CD 206, (**C**) CD 163, (**D**) TGF b, (**E**) IL6, (**F**) TNF, following 24 and 48 hours of lactate exposition, analyzed by Real time PCR. The calculated value of 2^-ΔΔCt^ in untreated controls is 1. Immunocytochemistry analysis of (**G**) iNOS and (**H**) Arg1, following 72 hours of lactate treatment. Data are expressed as mean ± SD of at least four independent experiments. (*p<0.05; **p<0.005; ***p<0.001; ****p<0.0001). Scale bars in (**G** and **H**) 10 μm.


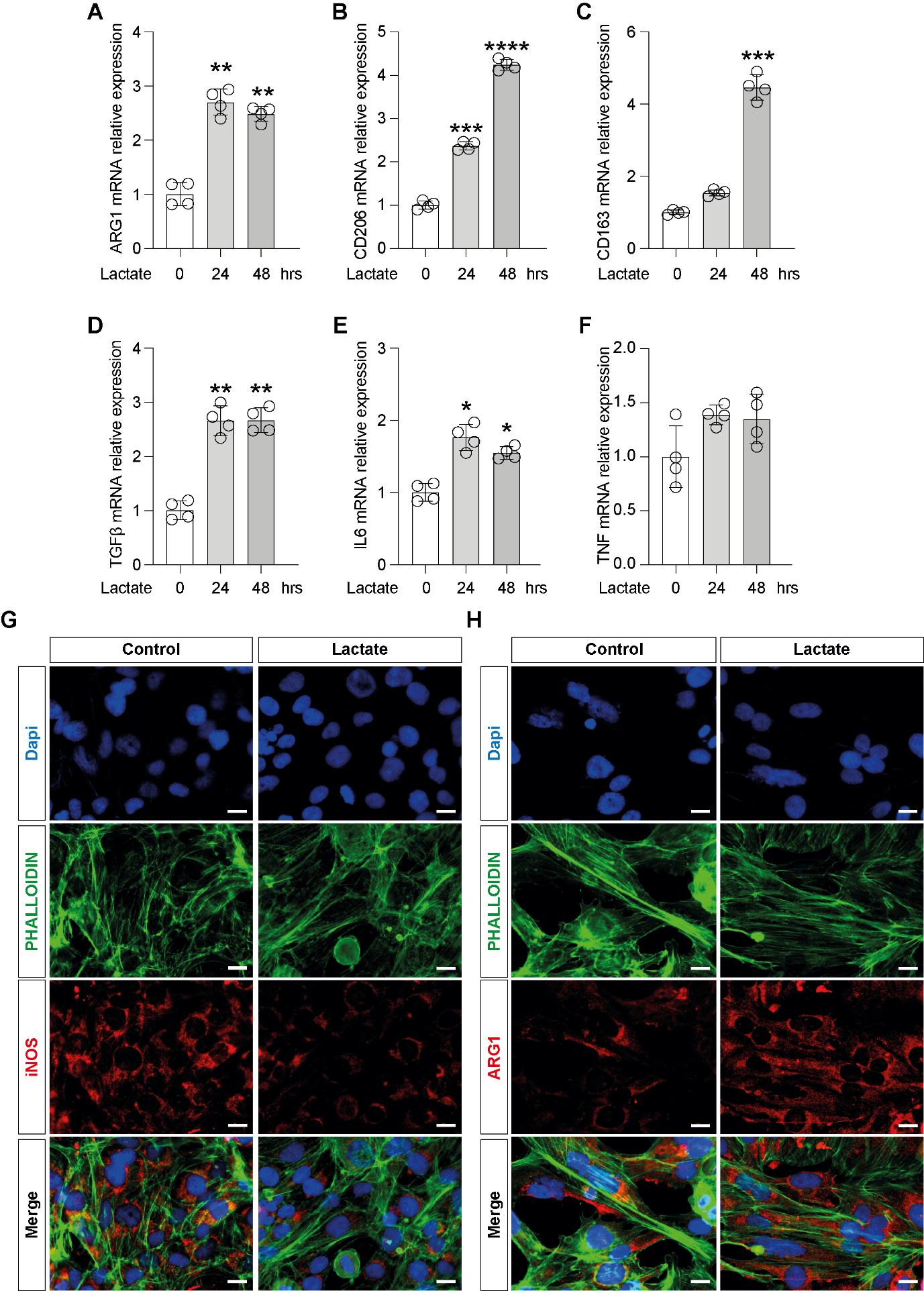

Supplement: Supplementary file 1 — Supplementary file1 (DOCX 2161 KB) [file 262_2022_3215_MOESM1_ESM.docx]
