# Supplementary material for: Lactate modulates microglia polarization via IGFBP6 expression and remodels tumor microenvironment in glioblastoma
Source: Cancer Immunol Immunother. 2022 Jun 3;72(1):1–20. doi: 10.1007/s00262-022-03215-3 (PMC9813126; doi:10.1007/s00262-022-03215-3)
Supplement: Supplementary file 2 — Supplementary file2 (DOCX 3226 KB) [file 262_2022_3215_MOESM2_ESM.docx]

**SUPPLEMENTARY MATERIALS**

Lactate modulates Microglia polarization via IGFBP-6 expression and remodels tumor microenvironment in Glioblastoma

**Table S1**. Relative mRNA expression levels of TNF, IL1B, ARG1, CD206 and CD163.

|  | **Control** | **Medium**  **U-87MG**  **Untreated** | **Medium**  **U-87MG**  **+ IGFBP-6** | **Control** | **Medium A-172**  **Untreated** | **Medium A-172 + IGFBP-6** | **Control** | **Medium U-251**  **Untreated** | **Medium U-251 + IGFBP-6** |
| --- | --- | --- | --- | --- | --- | --- | --- | --- | --- |
| **TNF** | 1,004 ± 0,101 | 0,444 ± 0,038 | 0,062 ± 0,012 | 1,004 ± 0,101 | 0,410 ± 0,054 | 0,157 ± 0,023 | 1,004 ± 0,101 | 0,101 ± 0,010 | 0,113 ± 0,005 |
| **IL1b** | 1,002 ± 0,064 | 4,610 ± 0,224 | 9,712 ± 1,317 | 1,002 ± 0,064 | 2,080 ± 0,451 | 4,721 ± 0,529 | 1,002 ± 0,064 | 15,662 ± 1,18 | 32,186 ± 4,32 |
| **ARG1** | 1,005 ± 0,111 | 4,370 ± 0,272 | 6,836 ± 0,552 | 1,005 ± 0,111 | 2,459 ± 0,496 | 12,047 ± 2,34 | 1,005 ± 0,111 | 1,606 ± 0,098 | 3,375 ± 0,379 |
| **CD206** | 1,004 ± 0,099 | 2,414 ± 0,139 | 3,952 ± 0,241 | 1,004 ± 0,099 | 1,375 ± 0,133 | 2,238 ± 0,439 | 1,004 ± 0,099 | 1,076 ± 0,059 | 1,148 ± 0,100 |
| **CD163** | 1,002 ± 0,068 | 1,612 ± 0,118 | 1,804 ± 0,184 | 1,002 ± 0,068 | 1,999 ± 0,442 | 9,846 ± 3,020 | 1,002 ± 0,068 | 1,312 ± 0,134 | 0,761 ± 0,053 |

**Supplementary Figure 1.**


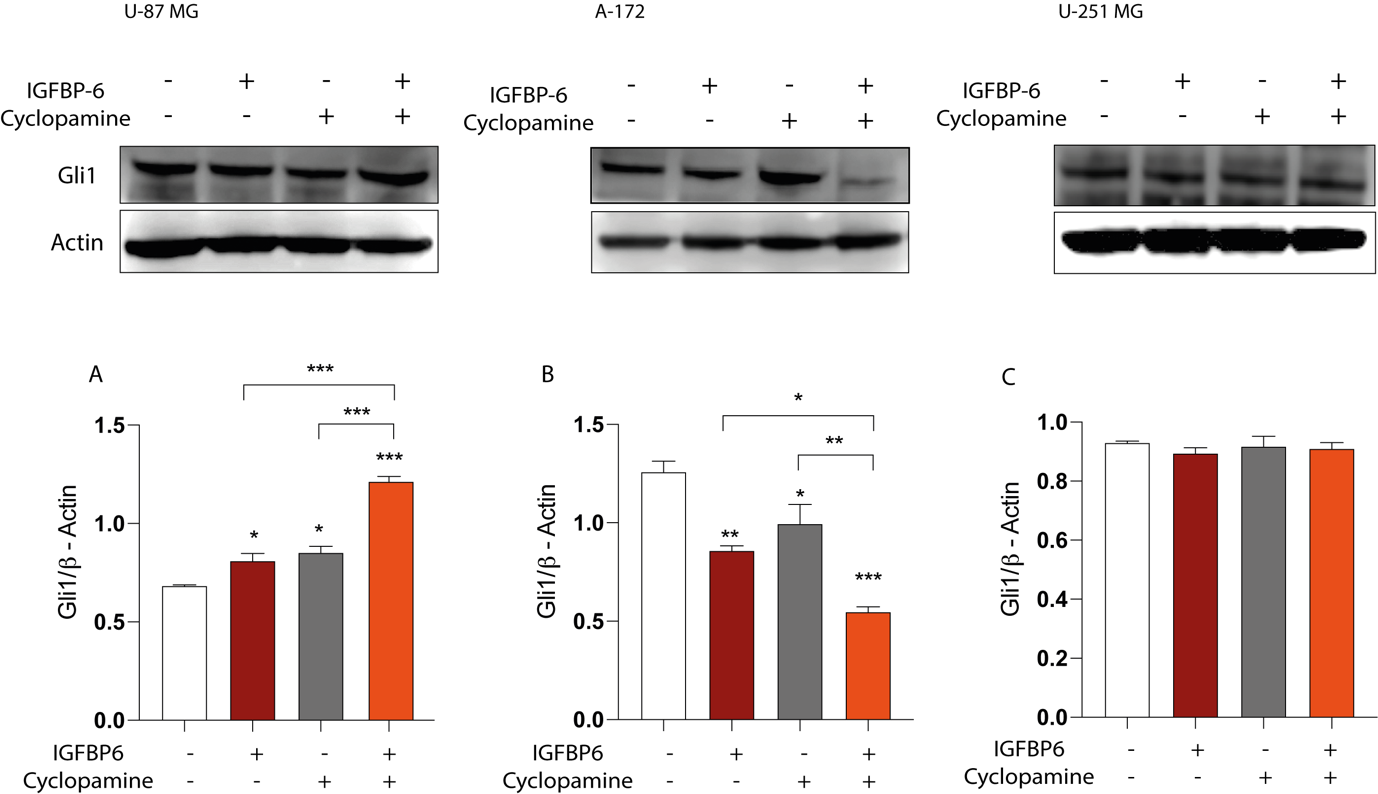


**Figure S2. Effect of IGFBP-6 and cyclopamine in GLI1 protein expression in Glioblastoma cell lines.** Evaluation of GLI1 protein expression after IGFBP-6 and cyclopamine treatment in U-87 MG (A), A-172 (B) and U-251 MG (C) cell lines. Data are expressed as mean ± SD of at least four independent experiments. (*p<0.05; **p<0.005; ***p<0.001; ****p<0.0001).
